# A Conversation with Nasser Altorki, MD, David B. Skinner, M.D. Professor of Thoracic Surgery, Weill Cornell Medicine

**DOI:** 10.1017/cts.2024.627

**Published:** 2024-11-08

**Authors:** Clinical Research Forum

**Affiliations:** Clinical Research Forum, Washington DC, USA

## Top 10 Clinical Research Achievement Awards Q & A

This article is part of a series of interviews with recipients of Clinical Research Forum’s Top 10 Clinical Research Achievement Awards. This interview is with Nasser Altorki, MD, David B. Skinner, M.D. Professor of Thoracic Surgery, Weill Cornell Medicine. Dr. Altorki is an internationally renowned thoracic surgeon with expertise in minimally invasive lung and esophageal surgery. His professional interests include lung cancer immunotherapy and prevention, esophageal diseases, and clinical trials in lung and esophageal cancer. Dr. Altorki received a 2024 Top 10 Clinical Research Achievement Award for Lobar or Sublobar Resection for Peripheral Stage IA Non-Small-Cell Lung Cancer. *The interview has been edited for length and clarity.*

## What drew you to the field of thoracic surgery?

When I was in medical school, I enjoyed two fields very much: cardiology and cardiothoracic surgery. I eventually gravitated toward surgery because as a surgeon, I not only get to understand the disease, but I can actively *do* something about it. I like taking action and the reward in surgery is more immediate than in other fields. Solving problems has always appealed to me – and the more complex the problem, the more I want to solve it.

## How did you get involved in clinical research?

One may be interested in research but the dilemma, of course, is that if one is a busy clinician, there’s just too little time for it. You can’t do research as a hobby. You have to make sure you’re asking the right question and then really dedicate yourself to answering it. That’s what happened with the trial that won the award. It was research that was in my lane.

## What was the central question of the award-winning research?

The question we asked was pretty straightforward. We wanted to know if, for certain lung cancer patients who have small tumors, we are doing too much by surgically removing the entire lung lobe. Lobectomy was established as the standard of surgical care for these patients based on a randomized trial done in the 1980s. But over time, many in the field were growing unsatisfied with that approach, mostly because advances in imaging and staging methods allow us to detect smaller and earlier tumors. Our goal was to repeat the previous trial but use more sophisticated imaging and a larger sample size, so we could have more confidence in the results.

## Essentially, you wanted to test the standard of care?

Yes, and at first, when I took the idea to the Cancer and Leukemia Group B (now part of the Alliance for Clinical Trials in Oncology), which is funded by the National Cancer Institute (NCI), I was 100% sure that the answer would be no, that they would not support this clinical trial. This was back in 2005, and I thought maybe there hadn’t been enough time since the trial that was done in the 80s (and reported in the 90s). But they did not say no and we were able to get the funding.

## What other obstacles did you have to overcome?

Doing a surgical randomized trial is difficult, especially when it involves an illness like cancer. Patients, in many cases, have already made up their minds about their treatment plans. So enrolling people took a while, as did getting surgical centers to join us. But eventually, we got everybody rowing in the same direction, and we ended up recruiting patients from 83 academic and community-based institutions in the USA, Canada, and Australia. We finished the accrual in 2017 and then because it had the potential to be a practice-changing trial, we had a five-year follow up, which took us to 2022, when we got the results.

## What was the result?

In the patient population we tested, which was people with peripheral non-small-cell lung cancer (NSCLC) with a tumor size of 2 cm or less and pathologically confirmed node-negative disease in the hilar and mediastinal lymph nodes, sublobar resection was not inferior to lobectomy with respect to disease-free survival. No substantial difference was seen between the two groups in the incidence of locoregional or distant recurrence. In addition, overall survival was similar with the two procedures. When I saw those results, it was such a great moment. It was so gratifying to see the results and know that it was going to help so many patients.

## How will patients benefit?

Obviously, the lung is a vital organ. It’s also one that does not regenerate, so once it’s gone, it’s gone. Most of the cancer patients I treat are former smokers or they’re older patients with other co-morbidities. If we can offer them the same cure rate without surgically removing a large amount of the lung, that is a win.

## Have thoracic surgeons begun to change the way they treat patients based on these results?

Yes. When you come up with a result that challenges 40 years of tradition, the first reaction tends to be skepticism, and we did get a little of that initially. But surgeons are scientifically minded and once they internalized the data, they came around. Some of the procedures for a sublobar resection are actually more complicated than the ones for lobectomy, so there’s been some learning, too. But people are stepping up to the plate. They’re learning and teaching it to the next generation. It took a little time, but already this has become mainstream.

## Sounds like a textbook example of clinical research improving clinical practice

Yes, and it shows why we need to have more support for clinical research. Without it, medicine would never advance. We forget that we have several responsibilities when we go into academic medicine. The first and most important responsibility is to our patients. That’s why we go to medical school. But we have two other commitments, as well. We have a teaching mission, which is training the next generation. And then we have an academic mission, meaning we need to try and move the field forward for the future. To do all three requires a lot of support and a lot of funding. That’s why collectively as a society we need to commit more resources to these types of studies, the ones that only the NCI can do. They are needed to improve clinical practice.

## What advice do you have for people starting their careers in clinical research?

It took me a long while but eventually, I think I’ve found the “secret sauce” to success. The first key ingredient, as I’ve mentioned a few times already, is funding, especially philanthropic funding. Sure, grants from public resources are important, but they’re also really hard to get because the process is so competitive. Grateful patients support research, so you need to raise money that way and then put it to good use. The second key ingredient is team science. You have to understand that nothing worthwhile is worth doing alone, nor can it be done alone. That’s why team science is so important. I work with an incredible group of people that span a range of specialties – biochemistry, biology, pathology, and computational medicine – and we meet regularly, every week. For me, this is definitely the best way to do medical science. That’s because we feed off of one another. By that I mean, whenever I step into that into that meeting, I know that I’m going to learn something. Really, every week, I learn something. They learn something. And together, we come up with new ideas. This group has received multiple grants and that would never have happened if any of us were trying to do it alone. So you need money, a team science mindset, and a group of smart people around you, helping you ask the right questions.

